# Examining the role of staff and team communication in reducing seclusion, restraint and forced tranquilisation in acute inpatient mental health settings: protocol for the Communication and Restraint Reduction (CaRR) study

**DOI:** 10.1136/bmjopen-2025-102989

**Published:** 2025-11-05

**Authors:** Francesca Cibelli, Trisha Forbes, Rose McCabe, Janet E Anderson, Juanita Hoe, Syeda Tahir, Gary J McKeown, Benjamin Brew, Felicity Deamer, Mary Lavelle

**Affiliations:** 1 School of Health and Medical Sciences, City St George’s, University of London, London, UK; 2 School of Psychology, Queen’s University Belfast, Belfast, UK; 3 School of Translational Medicine, Monash University, Melbourne, Victoria, Australia; 4 Geller Institute of Aging and Memory, School of Medicine and Biosciences, University of West London, London, UK; 5 Independent Lived Experience Consultant, London, UK; 6 South Eastern Health and Social Care Trust, Lisburn, UK; 7 Institute for Forensic Linguistics, Aston University, Birmingham, UK

**Keywords:** MENTAL HEALTH, Adult psychiatry, Inpatients

## Abstract

**Abstract:**

**Introduction:**

Over 100 000 service users are admitted to acute mental health wards annually, many involuntarily. Wards are under incredible pressure due to high bed occupancy rates and staff shortages. In a recent survey, over 80% of mental health nurses reported experiencing aggression and violence within their role. National and international policy dictates that mental health ward staff manage incidents of aggression and violence using communication, known as de-escalation. However, de-escalation practice is variable, and there is little empirical evidence to underpin training. As such, there is still a reliance on more restrictive practices, including seclusion and physical restraint.

**Aim:**

The aim of this study is to identify the communication and organisational factors that characterise effective management of service users’ behaviour and distress in acute adult inpatient mental health wards, reducing the reliance on more restrictive practices (eg, seclusion and restraint).

**Methods and analysis:**

This observational study will be conducted on mental health wards in England. It will be comprised of three work packages (WPs).

1. A microanalysis of communication during de-escalation incidents from Body Worn Camera footage on wards (n=64)*,* to identify staff communication practices that lead to effective management of service users’ distress.
2. Ethnographic observations of ward routine practice, alongside interviews and questionnaires with staff and service users, to examine how challenging behaviour is anticipated, planned for and responded to on wards, and staff experiences and perceptions of this process.
3. Triangulation of the findings from WPs 1 and 2 to examine the relationship between approaches to aggression management and staff communication, exploring the similarities and differences within and between wards.

**Ethics and dissemination:**

Ethical approval for sites in England has been granted by the Wales Research Ethics Committee 3, REF 22/WA/0066. Findings will be disseminated through peer-reviewed journals, scientific conferences and service user and clinical networks.

**STRENGTHS AND LIMITATIONS OF THIS STUDY**

- The first study using body-worn cameras (BWCs) to capture real-time de-escalation interactions, allowing for an in-depth analysis of communication during these high-stakes interactions.
- Integrates multiple data sources (eg, video recordings, ethnography, interviews and self-report questionnaires) to develop a comprehensive understanding of aggression management on mental health wards. This will provide empirical evidence to inform future practice, policy and training, ultimately resulting in the avoidance of physically and psychologically traumatic outcomes for service users (eg, restraint, seclusion and forced medication) and staff (eg, injury and burnout).
- Study-specific, expert by experience advisory group informing on all aspects of the research.
- While BWC footage provides rich data, staff will control when BWCs are activated; the footage may only capture parts of an incident, potentially omitting important contextual information, such as triggers leading up to the escalation.
- Study sites are in an urban location in England, and the generalisability of the findings may be limited.

## Introduction

 Mental health services in the UK are currently under considerable pressure and extremely stretched. Since 2009, there has been a 30% reduction in mental health inpatient beds,^1 2^ and 91% of acute adult mental health wards frequently operate above the recommended 85% bed occupancy.^3^ In a recent national survey by the British Medical Association, over half (65%) of mental health nurses in the UK reported that on their last shift worked, there was a shortage of at least one member of staff.^4^ Bed and staff shortages mean that only the most acutely unwell service users are admitted to wards.

Conflict, including aggression and violence, is common in this challenging environment.^5^ Over 80% of nurses annually in England report directly experiencing service user aggression.^6^ Approximately 60 000 restraints occur annually in mental health wards in England, equating to six service users being restrained per hour.^7^ Mostly, staff manage service user aggression effectively through verbal and non-verbal communication, known as de-escalation. However, in approximately one-third of cases, de-escalation is not effective,^8^ resulting in the reliance on more restrictive practices including physical restraint (holding the service user to prevent movement), seclusion (isolation in a confined room) and forced tranquillisation (involuntarily injected with psychotropic medication).^8^

The human and financial cost of restrictive practice is high. Physical restraint has resulted in a service user’s death.^9^ Alongside physical harm, service users experience significant psychological distress, including trauma, fear, feeling ignored, disempowered and dehumanised.^9 10^ Traumatic effects are not limited to those with direct experience but are also experienced by those observing others being restrained.^11^ Service users who experience restrictive practices are more likely to display aggression again during their admission, have significantly longer inpatient admissions and have poorer therapeutic relationships with staff,^12^ which is a reliable predictor of prognosis in mental healthcare.^13^ Staff also experience physical injuries, anxiety and burnout, leading to poor staff retention.^6 14^

Nationally and regionally, the use of restrictive practices in mental health settings is recognised as a significant concern. The recent Darzi report highlighted the increase in restrictive practice nationally in recent years.^15^ This is particularly relevant in the current review of the Mental Health Act, while regional policies aimed at reducing the use of restrictive practice have recently been introduced, including the Mental Health Use of Force Act (Seni’s Law)^16^ in England and Wales, and the recent Regional Policy on the use of restrictive practices in Northern Ireland.^17^

Despite recognition of the need in policy and practice, there remains a lack of empirical evidence to underpin de-escalation training to support staff; there is no nationally agreed approach to best practice.^18^ De-escalation practice varies significantly by wards, even when service user populations remain similar.^19^,^21^ Studies exploring perceptions of effective de-escalation suggest good communication is critical^2022^,^24^; specifically, staff communication with service users should be respectful, compassionate, engaging and fostering a therapeutic relationship^23 25^; staff teams that are cohesive, reflective and display open communication (ie, feeling safe to discuss their approaches to service user aggression openly and honestly) have also been identified as important.^26 27^ These are quite high-level concepts. Identifying the specific behaviours that achieve these (eg, what behaviours reflect respectful and compassionate communication) would be a first step in providing tangible information to inform staff training and practice.

The introduction of body-worn cameras (BWC) to mental health wards across some National Health Service (NHS) Trusts to record staff management of service user aggression^28^ provides an opportunity to examine communication during de-escalation practice at a level of granularity, which has not previously been possible. BWC will be employed in the current study for this purpose.

Here we present a protocol for a study that will examine staff communication and organisational practices designed to prevent and respond to aggression and violence in acute adult inpatient mental health wards, identifying effective approaches that reduce the reliance on restrictive practices, including physical restraint, seclusion in a confined room or forced tranquilisation.

## Methods

### Study design and objectives

This is an observational mixed-methods study. It will be conducted in acute adult inpatient mental health wards (a minimum of five) across mental health trusts in England. Wards will be purposively recruited to balance gender and, where possible, acuity (based on the average number of recorded incidents per week). The diversity in ward gender, acuity and hospital trust will provide diversity in the sample, improving the generalisability of findings.

**Figure 1:**
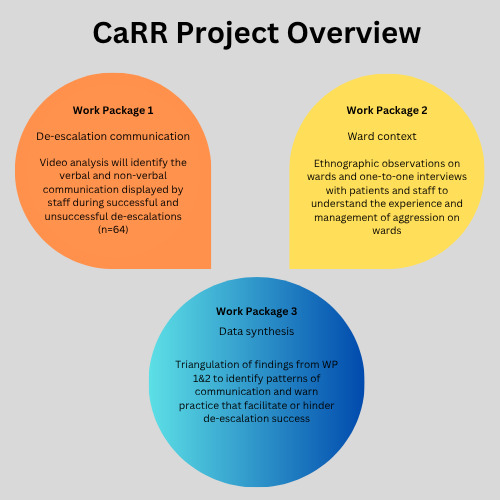
Project overview: relationship between WPs 1–3. CaRR, Communication and Restraint Reduction; WP, work package.

The study will be comprised of three work packages (WPs) (see figure 1 for a summary), which address the following objectives.

#### WP1: staff communication

- *Objective 1*—to identify the verbal and non-verbal communication of staff that is associated with effective de-escalation.

Video recordings of de-escalation events (n=64) will be recorded across participating wards. The verbal and non-verbal communication of staff will be analysed. The relationship between communication practices and de-escalation success will be examined statistically and qualitatively.

#### WP2: ward context and organisational approaches

- *Objective 2*—to observe how service user aggression is anticipated, discussed, planned for and responded to on wards, during routine shifts.
- *Objective 3*—to explore staff and service users’ experience of de-escalation and their perceptions of how aggression is responded to on their ward.

Non-participant ethnographic observations will examine how ward teams discuss, plan for and respond to service user aggression more broadly during a routine shift. This data will be contextualised with one-to-one semistructured interviews and standardised questionnaires with service users and staff (exploring their experience and perceptions). Interviews will be conducted face-to-face, where possible, at participating sites in a separate room to allow for privacy. However, interviews can be conducted on secure online platforms (eg, Teams) when face-to-face meetings are not possible.

#### WP3: data triangulation and synthesis

- *Objective 4*—to examine the relationship between approaches to aggression management and staff communication, exploring the similarities and differences within and between wards.

Findings from WPs 1 and 2 will be synthesised using a process of triangulation. The staff and team communication observed will be mapped across wards to examine the relationship between de-escalation communication and ward contextual features. This will provide a more comprehensive picture of the facilitators of aggression prevention and effective de-escalation.

### Inclusion and exclusion criteria

All healthcare professionals working on participating NHS wards will be eligible for participation in the study.

All service users involved in a de-escalation incident on the participating ward, and deemed by the ward lead to have the capacity to provide informed consent to be approached, will be eligible for inclusion. Service users who are younger than 18 years old, over 70 years old or do not have the capacity to consent will not be eligible for inclusion in the study. Service users who require an interpreter will also be excluded from the study due to the limited capacity of the research team and the critical role of communication in this study.

Recorded de-escalation incidents will only be included when the footage quality is sufficient to conduct detailed observational analyses of verbal and non-verbal behaviour; staff must be visible, or partially visible in footage, and voices should be audible. As the focus of the communication analysis is on staff, where service users are unable to provide informed consent, their image will be redacted from the footage prior to analysis so they are no longer identifiable in the video.

### Consent procedure

**Figure 2:**
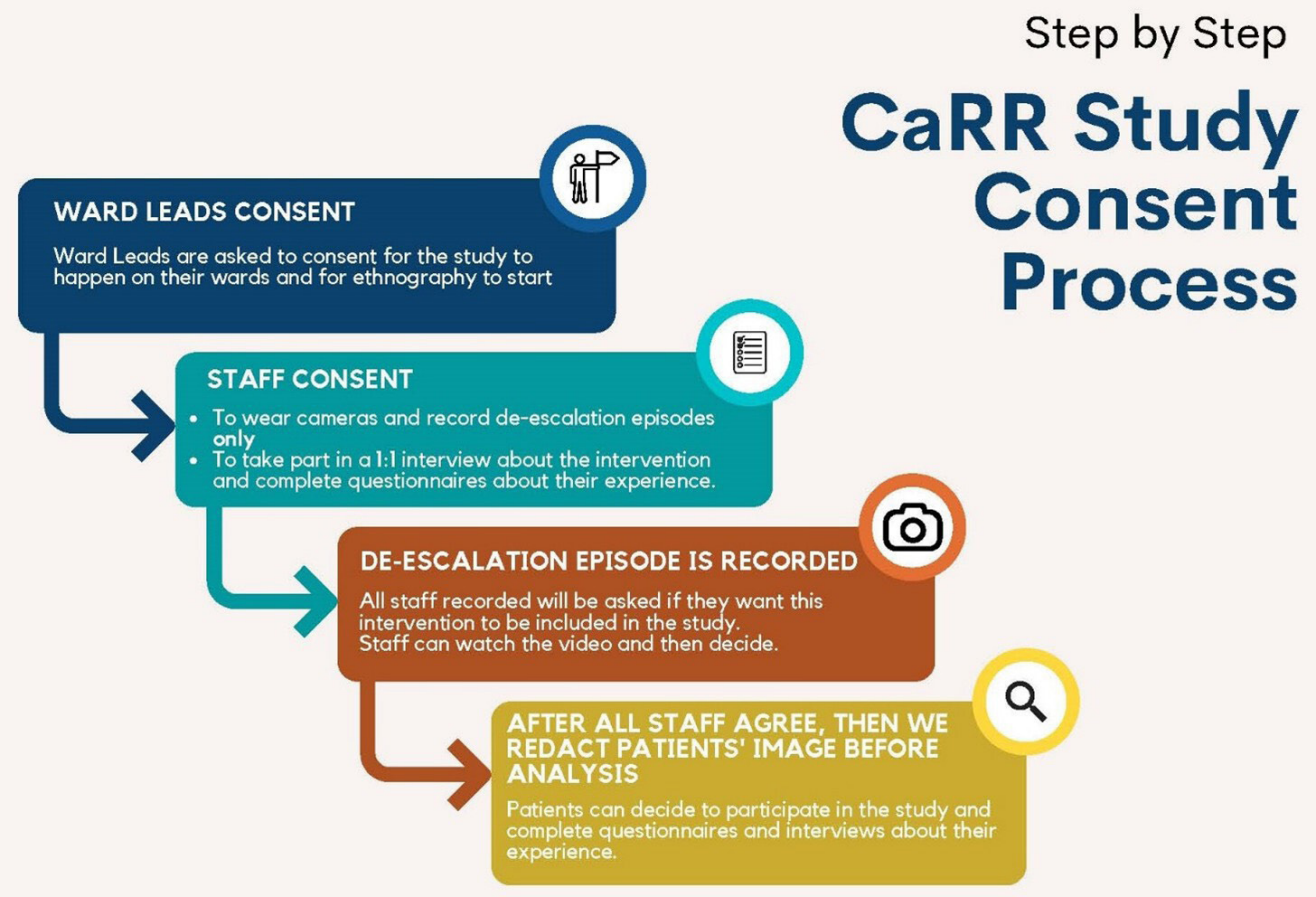
CaRR study consent process. CaRR, Communication and Restraint Reduction.

It is acknowledged that this is a highly sensitive and challenging topic. As such, a stepped consent procedure will be in place to ensure openness and transparency at all levels of study involvement (see figure 2).

### Study visibility

Before being approached for consent, service users and staff will be made aware that the study is happening due to several prestudy engagement events and the presence of flyers and posters on the ward. These will be developed in collaboration with experts by experience researchers, informing service users of the study and its purpose. The clinical researcher will be present on wards conducting ethnographic observations for several weeks prior to any consent being requested. Therefore, service users will be familiar with the researcher and the study and have an opportunity to ask questions.

#### Ward leads

First, ward leads will be approached and asked if they wish for their ward to participate in the study. They will be provided with a study information sheet and will have the study explained to them by members of the research team, and will have an opportunity to ask questions. If ward leads provide informed consent for their ward to participate in the study, they will be providing consent for BWCs to be used on their ward (if they are not used routinely) to record incidents of de-escalation, and for a researcher to conduct ethnographic observations of routine work on their ward.

#### Ward staff

To alleviate staff concerns about having their practice recorded, we will use a two-step consent process.

- Step 1—the study will be presented to staff, and information sheets will be provided alongside an opportunity to ask questions. Staff have the option to provide consent to participate in the study.
- Step 2—if a staff member is identified in a recorded incident that meets the inclusion criteria, they will be given an opportunity to watch their recorded de-escalations. At this point, staff can consent to the video being analysed or ask for it to be withdrawn. This step will happen for all staff in any video they are involved.

If any member of staff is recorded in the footage but does not play a key role in the de-escalation process (eg, in the background, not interacting with the service user), they will be redacted from the footage prior to analysis by a member of the research team.

#### Service users

It would be inappropriate and potentially unsafe to try to approach service users for consent at the time of a de-escalation incident. As such, service user consent to participate will only be sought retrospectively, not at the time of the incident. Service users who lack the capacity to provide informed consent will not be approached. Decisions on capacity will be made by ward leads. When requesting consent, service users will be approached at a time that the ward lead deems appropriate. Service users will be given a study information sheet (paper version), which they can keep. The researcher will verbally explain the study to them and explain that we are interested in analysing a specific incident that they were involved in previously on the ward. They will have an opportunity to ask questions and will be encouraged to do so. The researcher will answer any queries or questions service users have. Service users will be given 2 days to decide if they want to participate. The researcher will revisit the service user after 2 days to obtain their response. As ward populations are dynamic, the clinical researcher will work closely with ward leads to ensure service users who are eligible to participate are approached prior to discharge, where possible.

#### Contingency plan for recruitment challenges

Although the preference is to obtain consent from both service users and staff for analysis of the recorded de-escalation incidents, it is acknowledged that recruitment of service users who are involved in de-escalation incidents may be particularly challenging. The focus of this research is to examine staff communication, rather than the behaviour of service users. Therefore, as a contingency plan, where service user consent cannot be obtained, and staff have consented for the footage to be included, the service user’s image will be redacted from the footage prior to analysis. This will involve pixellating the service user’s image so they are no longer recognisable. Image redaction from the video will be carried out by a member of the research team.

## Patient and public involvement

Experts by experience have informed all stages of the study development. The study design and original funding application were presented to, and shaped by, the established Service User and carer Group Advising on Research group in City University of London. An expert by experience co-investigator has been involved from the outset. A dedicated expert by experience group for the study has also been set up, involving 10 people with direct experience of restrictive practice while detained in mental health hospitals and their carers across the UK. This expert group will be involved in data collection, analysis, interpretation and dissemination of the study. This group will also facilitate the translation of findings into practice.

## Data collection

### Audiovisual recordings of de-escalation (BWCs)

In participating wards that routinely use BWCs as part of their practice, ward staff will be asked to record *all* interactions when de-escalating a situation involving a service user or when they think that a service user’s behaviour is likely to escalate.^28^ In participating wards where BWCs are not used routinely, BWCs will be introduced for the purpose of this study. Staff on these wards will receive camera training. The practice of recording on these wards will be identical to wards that use cameras routinely. All cameras use a secure recording system, similar to those used by the police.^28^

On each ward, de-escalation incident recordings are uploaded at the end of every shift to a secure cloud-based server hosted by the BWC supplier (ie, Reliance Protect | Body WornCameras. All recordings will only be available to review by the research team and Trust study collaborators via access to the secure portal using a username and password. De-escalation recordings of poor quality will be immediately deleted. Recordings of sufficient quality for analysis will be assessed to see if they meet the inclusion criteria. If eligible for inclusion, consent from the service user and staff involved will be sought. If consent is provided, de-escalation recordings will be included for analysis. Other service users, or staff members captured in the background of the video who are not directly involved in the de-escalation incident, will be redacted from the video footage so their image is pixelated and not identifiable in the footage prior to analysis.

Expert guidance was followed on sample size calculations^29^ by calculating the sample size required to detect a clinically significant effect of prosocial behaviour on de-escalation success. Information from our prior study^8^ was used to estimate the likely distribution of prosocial behaviour in our sample.

On the basis of our sample size calculations, we propose to sample n=64 de-escalation interactions. Powersim module in STATA V.27 was used to estimate the sample size required to detect a doubling of successful de-escalation attempts in interactions with high levels of prosocial behaviour versus those with low prosocial behaviour, at 80% power at alpha=0.05, assuming a ratio of 5:3 high prosocial:low prosocial, as found in our previous study.^8^ This suggested n=64 was the minimum sample size required to detect this difference.

### Ethnographic observations

Ethnographic observations will examine how ward teams discuss, plan for and respond to service user aggression during routine shifts. Observations will involve shadowing staff and having brief discussions with them about their work in situ. Observations will be documented as field notes.

Observations will explore:

- How and when staff discuss or plan for service user aggression on wards during routine shifts, including both formal (eg, safety huddle) or informal discussions (eg, chat between nurses)
- Contextual factors that might influence those discussions (eg, hierarchy, trust and staff mix)
- How staff respond to an incident of aggression, including what happens before and after the cameras are recording
- The impact of restrictive practice on the ward environment and relationships, and vice versa

### Interviews with service users and staff

One-to-one semistructured interviews with participating service users and staff will be conducted. A minimum of 10 interviews per group (staff or service user) will be conducted. All interviews will be recorded and transcribed. Interview schedules will be informed by ethnographic observations.

Purposive sampling will be used to recruit interview participants with direct experience of de-escalation or managerial staff with responsibility for ensuring patient and staff safety.

Service user interviews will be conducted with service users who consented to the analysis of their recorded de-escalation incident. As the study will involve two male and two female wards, each gender will be equally represented. We aim to recruit a representative sample of participating wards in terms of participant ethnicity. Our previous study identified that the majority of patients experiencing de-escalation were white (68%).^8^ As such, we will aim to recruit at least 6 patients (38% of our sample) who are from Black, Asian and Minority Ethnic (BAME) backgrounds across the sample, who have experienced both successful (n=3) and unsuccessful de-escalation (n=3). Interviews will focus on service users’ experience of how staff responded to them during their de-escalation incident, and what, if anything, they feel could have been done differently if the situation happened again. Service users will also be asked about their observations and perceptions of how their aggression is managed on their ward.

Purposive sampling for interviews will be used to try to gain a range of perspectives from staff working at different hierarchical levels and contract types within the wards, including ward leads, qualified nursing staff, unqualified healthcare assistants and temporary/agency staff. Where possible, we will interview two males and two females from each post across the four wards. We will attempt to have 50% of our sample comprised of staff from BAME backgrounds, and we will try to get diversity in staff years of experience. Staff interviews will focus on staff experience of aggression management on their ward, their perception of safety, support and trust within the ward team and the impact on their relationship with service users. Interview schedules will be refined based on the ethnographic observations.

### Quantitative measures

#### Staff measures

A questionnaire battery will be administered to staff to collect the following information:

- *Demographic information and experience*—age, gender, ethnic group, staff role, experience working in mental health (in years) and aggression management training completed.
- *Violence at work*—assessed using three related measures^30^: (1) the Direct experience of Violence at Work scale,^30^ an eight-item questionnaire measuring the occurrence and frequency of violent events experienced by staff (internal consistency α=0.65); (2) the Vicarious experience of Violence at Work (VVW) scale,^30^ an eight-item questionnaire, measuring the violence witnessed by staff at work (internal consistency α=0.88); (3) the Fear of Future Violence at Work scale,^30^ a 10-item questionnaire measuring the degree to which staff fear becoming a victim of future violence at work (internal consistency α=0.94).
- *Staff attitudes towards aggression management*—assessed using The Management of Aggression and Violence Attitude Scale Likert version (MAVAS-L),^31^ a 27-item questionnaire containing statements about the causes of aggression and approaches to aggression management, participants are asked to rate, on a five-point Likert scale, their agreement with each statement (strongly agree–strongly disagree). The scale shows good reliability (*r*=0.89).
- *Staff health and well-being*—assessed using two scales: (1) The Clinical Outcomes in Routine Evaluation 10,^32^ a 10-item questionnaire measuring staff psychological distress in the past week (internal reliability α=0.90; concurrent validity *r*=0.90); (2) the Short Form Health Survey,^33^ a 12-item survey measuring the impact of health on everyday life (internal consistency α >0.80).
- *Ward atmosphere*—assessed using two questionnaires: (1) work-related affect scale,^34^ a six-item questionnaire, asks staff members to indicate how often the job made them feel—uneasy, contented, depressed, relaxed, enthusiastic or tense in the previous year, from never to always on a 7-point Likert scale (internal consistency α=0.87); (2) the Good Milieu (GM) Index,^35^ a five-item questionnaire assessing staff members’ satisfaction with their ward (concurrent validity *r*=0.88).

#### Service user measures

Service users’ age, gender, ethnic group, formal diagnosis, history of violence and whether this is a first admission will be extracted from their clinical notes by the researcher after service users have given formal consent for this. This will be obtained and processed according to the Data Protection Act 2018 and UK General Data Protection Regulation (GDPR).

A questionnaire battery including three questionnaires will be administered to service users when they provide their consent to participate. Questionnaires will examine the following:

- *Service users’ experience of communication with staff during de-escalation*—assessed using the Patient Experience Questionnaire.^36^
  This is a 12-item questionnaire with three subscales where service users rate their (1) experience of communication, (2) barriers to communication and (3) emotions immediately after the interaction (scale internal consistency α >0.70).
- *Service users’ attitudes towards aggression management*—assessed using MAVAS-L version,^31^ a 27-item questionnaire where service users rate their agreement with each item on a 5-point Likert scale (strongly agree–strongly disagree). The scale shows good reliability (*r*=0.89).

*Ward atmosphere*—assessed using the GM Index,^35^ a five-item questionnaire to assess service users’ satisfaction with their ward (concurrent validity *r*=0.88).

#### Video rating measures

The English Modified De-escalating Aggressive Behaviour Scale (EMDABS)^37^ will be used to examine the quality of the de-escalation performance from video. The EMDABS is a tool designed to evaluate the quality of the de-escalation performance. It is comprised of seven categories on which the de-escalation performance is rated (eg, valuing the client, remaining calm and reducing fear). Good inter-rater reliability (Intraclass Correlation Coefficient=0.75) and internal consistency (α=0.90).

## Data analysis

### WP1—analysis of de-escalation incidents from video

Recorded incidents of de-escalation will be transcribed and annotated using the language annotation software ELAN (https://tla.mpi.nl/tools/tla-tools/elan/). ELAN allows multiple video recordings to be synchronised and analysed simultaneously, which is necessary for BWC footage where there are multiple recordings of one event. It also facilitates microanalysis of verbal and non-verbal communication.

Pilot data collection revealed several important considerations for the analysis of video footage. BWC footage is limited, and the quality of recording for each incident may vary (eg, people occluded, camera moving). It is expected that the average duration of recorded incidents will be approximately 7 min, ranging from 3 to 20 min. The verbal and non-verbal communication displayed by staff will be identified and examined at a microlevel, in sequence, to reveal the relationship between staff communication and de-escalation success.

#### Communication analysis

Speech and non-verbal communication present in the recorded footage will be annotated using both an inductive and deductive approach.

Speech will be transcribed by a member of the research team. Conversation analysis techniques will be used to conduct an in-depth sequential analysis of communication.^38^ This will involve behavioural annotation of a range of communication practices at a microlevel. An example of one such practice is conversational repair. During conversation, we use a practice called repair to amend our own speech (self-repair) and others’ speech (other-repair) to develop shared understanding with those we are talking to. The amount of repair present in speech is indicative of the effort invested in developing a shared understanding,^39^ and has been linked to therapeutic relationships and satisfaction in mental healthcare.^40^

Deductive analysis of non-verbal communication will be guided by the Ethological Coding System for Interviews,^41^ a validated observational scale which categorises 37 non-verbal behaviours (ie, head, body, arms, face and eye gaze) in terms of their communicative meaning (ie, prosocial behaviour, socially avoidant behaviour, displacement behaviour—signalling tension, assertion and relaxation). Other non-verbal behaviours present in the video data will be identified and analysed inductively.

Communication between staff members will be analysed using the Temporal Observational Analysis of Teamwork (TOAsT) in the healthcare framework.^42^ TOAsT has been developed for use in mental health settings by Lavelle and Anderson. This framework contains 23 observable communication behaviours, grouped based on their function (eg, delegating, information gathering), which cluster into five overarching teamwork domains (eg, leadership) (Cohen’s Kappa=0.84, SE=0.03).

#### De-escalation success

De-escalation success is nuanced and may not merely be the absence of restrictive practice; as such, it will be operationalised in three ways:

1. The use of restrictive practice (physical restraint, forced medication or seclusion) will be annotated in the video footage.
2. The quality of the de-escalation performance will be evaluated using the EMDABS^37^ from the video. The EMDABS is a tool designed to evaluate the quality of the de-escalation performance. It is comprised of seven categories on which the de-escalation performance is rated (eg, valuing the client, remaining calm and reducing fear). Good inter-rater reliability (ICC=0.75) and internal consistency (α=0.90).
3. The points of escalation will be identified based on the service users' verbal and non-verbal behaviour. Point of escalation is a departure from the service user’s baseline behaviour as a consequence of a stimulus (internal or external) which can take the form of increased agitation or distress, this is visible as: increased tone of voice, increased pressure of speech, swearing, crying, increased and erratic movements, verbal or physical aggression towards others or self. Multiple points of escalation may be evident during one recorded interaction.

#### Statistical analysis

Spearman’s Rho correlations will investigate the relationships between markers of de-escalation success (use of restrictive practice; de-escalation quality (EMDABS); occurrence of escalation/de-escalation) and staff communication (verbal, non-verbal and team communication) practices.

Significant relationships will be further investigated using Generalised Estimating Equation (GEE) models. GEE will allow for clustering of de-escalation incidents by the ward on which they occurred. This adjusts for potential correlations between incidents that happen on the same ward. In each model, the predictors (ie, independent variables) will include: verbal and non-verbal communication and team behaviour. Staff and service user variables found to be associated with de-escalation success will also be included in the GEE models where appropriate.

### WP2—analysis of ethnographic observations and interview data

Ethnographic observations will be typed up by the researcher. Interviews will be automatically transcribed on Microsoft Teams and checked by the researcher for accuracy. Transcriptions and ethnographic field notes will be pseudoanonymised and imported into the qualitative software NVIVO (www.qsrinternational.com/nvivo/) for storage and analysis. At this stage, the original audio files will be destroyed. Reflexive thematic analysis will be conducted as described by Braun *et al*.^43^ This analysis will employ both an inductive and deductive approach.

### WP3—triangulation of findings from video data and ethnographic observations

The findings from WPs 1 and 2 will be synthesised using a process of triangulation, described by O’Cathain and colleagues.^44^ Findings from WPs 1 and 2 will be organised into a matrix. Findings from each data collection method are entered along columns, while participants grouped by wards will be entered as rows.

The matrix allows identification of points where findings derived from the three data collection methods agree (convergence), offer complementary information on the same issue (complementarity) or disagree (dissonance/discrepancy). This process results in a ‘convergence coding matrix’ with themes that cut across WPs.^44^ Findings will be compared between wards of different genders, locations and acuities; commonalities and differences will be identified. This triangulation process will provide a comprehensive picture of aggression management in acute mental health ward settings, including the barriers and facilitators to success.

In-depth descriptive analyses of individual de-escalation events will map the staff and team behaviours that co-occur in successful and unsuccessful de-escalation events (WP1). The behavioural patterns identified will be considered in the context of the qualitative themes identified on the ward where they took place and, where available, the perceptions and experiences of patients and staff involved in the incident (eg, when one-to-one interviews have been conducted (16 patients and 16 staff) (WP2). The patient factors will also be considered (eg, diagnoses, history of violence and whether it was a first admission). Findings from this triangulation will identify patterns of communication that facilitate or hinder de-escalation success, which could be considered at a staff, team and organisational level.

## Ethics and dissemination

This study has been subject to a peer review as part of the NIHR RfPB funding application process. Ethical approval for data collection in sites in England has been granted by the Wales Research Ethics Committee 3, REF 22/WA/0066.

### Data storage

Audiovisual recordings of de-escalation incidents will be recorded using discrete BWCs (eg, https://www.relianceprotect.co.uk/body-worn-cameras). This software is used by the police and other healthcare trusts, ensuring secure, encrypted data capture and cloud-based storage. The data is collected on encrypted devices and automatically uploaded to a secure server controlled by the camera provider. The server is only accessible to participating ward leads and the research team. Encrypted devices will be in a secure, locked location when not in use.

Recorded incidents where service users and staff have provided informed consent for inclusion in the study will be stored in a password-locked folder on secure servers in the Host University. Data will only be accessible by members of the research team for analysis purposes.

### Dissemination

Anonymised study findings will be disseminated via peer-reviewed journals and national and international conferences. Where consent has been provided by participants (service users and staff), anonymised quotes may be used in publication. Publication authors will be members of the research team. Findings will also be presented on the project website (CaRR), Involve and Trust newsletters. Participants who have consented will have an opportunity to receive report summaries of the findings via the study website. The study will be registered on the National Institute for Health and Care Research (NIHR) Clinical Research Network (CRN) portfolio and the lead investigator’s Researchfish account.

A multidisciplinary de-escalation working group, developed as part of the study, will facilitate the translation of findings into policy, training and practice. The first step in this work will be the delivery of research into practice workshops at participating wards.
