# Supplementary material for: Examining the role of staff and team communication in reducing seclusion, restraint and forced tranquilisation in acute inpatient mental health settings: protocol for the Communication and Restraint Reduction (CaRR) study
Source: BMJ Open. 2025 Nov 5;15(11):e102989. doi: 10.1136/bmjopen-2025-102989 (PMC12593446; doi:10.1136/bmjopen-2025-102989)
Supplement: Supplementary file 1 [file bmjopen-2025-102989.reviewer_comments.pdf]

PEER REVIEW HISTORY

BMJ Open publishes all reviews undertaken for accepted manuscripts. Reviewers are asked to complete a checklist review form and are provided with free text boxes to elaborate on their assessment. These free text comments are reproduced below.

ARTICLE DETAILS

Title (Provisional)

Protocol for the Communication and Restraint Reduction (CaRR) Study: An observational study examining the Role of Staff and Team Communication in Reducing Seclusion, Restraint and Forced Tranquilisation in Acute Inpatient Mental Health Settings

Authors

Cibelli, Francesca; Forbes, Trisha; McCabe, Rose; Anderson, Janet E.; Hoe, Juanita; Tahir, Syeda; McKeown, Gary J.; Brew, Benjamin; Deamer, Felicity; Lavelle, Mary

---

This article was not externally reviewed at BMJ Open. Protocols that have been independently assessed prior to submission to BMJ Open are usually fast-tracked to publication on the grounds that further substantial changes will not be possible. This independent assessment will usually be external, independent review for both a substantial grant award from a non-commercial or government funder and ethics approval.
